# *Escherichia coli*-based production of recombinant ovine angiotensinogen and its characterization as a renin substrate

**DOI:** 10.1186/s12896-016-0265-x

**Published:** 2016-04-07

**Authors:** Shinji Yamashita, Naoya Shibata, Akiyoshi Boku-Ikeda, Erika Abe, Ayumi Inayama, Takashi Yamaguchi, Ayano Higuma, Kaoru Inagaki, Tomoyo Tsuyuzaki, Satoshi Iwamoto, Satoshi Ohno, Takashi Yokogawa, Kazuya Nishikawa, Kazal Boron Biswas, A. H. M. Nurun Nabi, Tsutomu Nakagawa, Fumiaki Suzuki, Akio Ebihara

**Affiliations:** United Graduate School of Agricultural Science, Gifu University, 1-1 Yanagido, Gifu, 501-1193 Japan; Graduate School of Applied Biological Sciences, Gifu University, 1-1 Yanagido, Gifu, 501-1193 Japan; Faculty of Engineering, Gifu University, 1-1 Yanagido, Gifu, 501-1193 Japan; Faculty of Applied Biological Sciences, Gifu University, 1-1 Yanagido, Gifu, 501-1193 Japan; Department of Biochemistry and Molecular Biology, University of Dhaka, Dhaka-1000, Bangladesh

**Keywords:** Angiotensinogen, Renin, Angiotensin, Hypertension, Plasma renin concentration, *E. coli*, Recombinant protein production, Auto-induction

## Abstract

**Background:**

Angiotensinogen (ANG) is a macromolecular precursor of angiotensin, which regulates blood pressure and electrolyte balance. ANG is specifically cleaved by renin, an aspartic protease, to initiate the angiotensin-processing cascade. Ovine ANG (oANG) from sheep plasma has been shown to be a better substrate for human renin, and it has been used in clinical renin assays. To expand the availability of oANG, we aimed to produce milligram levels of recombinant oANG using an *Escherichia coli* expression system.

**Results:**

When recombinant oANG was expressed from a T7 promoter in various *E. coli* strains at 37 °C, it accumulated in the insoluble fraction. However, by expressing oANG at 37 °C from a *tac* promoter, which has weaker transcriptional activity than a T7 promoter, we significantly elevated the ratio of soluble to insoluble recombinant oANG. Using a novel culturing system and auto-induction culture medium, we purified *tac*-expressed recombinant oANG to homogeneity, with a yield of 4.0 mg per liter of culture. Based on size-exclusion gel filtration analysis and dynamic light scattering analysis, the resulting purified oANG is a monomer in solution. The circular dichroism spectrum of *E. coli*-expressed recombinant oANG was similar to that of oANG expressed in CHO cells. Differential scanning fluorimetry showed that both preparations undergo a two-state transition during thermal denaturation, and the melting temperatures of recombinant oANG expressed in *E. coli* and CHO cells were 49.4 ± 0.16 °C and 51.6 ± 0.19 °C, respectively. The *K*_m_ values of both oANG preparations were similar; the *k*_cat_ value of *E. coli*-expressed recombinant oANG was slightly higher than that of CHO-expressed oANG.

**Conclusions:**

Recombinant oANG expressed in *E. coli* functions as a human renin substrate. This study presents an *E. coli*-based system for the rapid production of milligram quantities of a human renin substrate, which will be useful for both fundamental and clinical studies on renin and hypertension.

**Electronic supplementary material:**

The online version of this article (doi:10.1186/s12896-016-0265-x) contains supplementary material, which is available to authorized users.

## Background

Angiotensinogen (ANG) is a 452-amino acid plasma glycoprotein. This protein is specifically cleaved by the aspartic protease renin (EC 3.4.23.15) to release the N-terminal decapeptide angiotensin I (AI). AI is further hydrolyzed by angiotensin-converting enzyme (EC 3.4.15.1) to produce the octapeptide angiotensin II, which regulates blood pressure and electrolyte balance [1]. ANG-deficient mice [2] and renin-deficient mice [3] exhibit profound hypotension, and no detectable AI was found in the plasma of mice with either knockout mutation [2, 3]. These in vivo studies demonstrate the biological importance of enzymatic cleavage of ANG by renin.

Human renin has a much higher affinity for and shows a much greater velocity with ovine ANG (oANG) than human ANG [4–6]. Native oANG, purified from sheep plasma, has been shown to be a better substrate for human renin, and it has been used in clinical renin assays [6]. The purification process for native oANG has been established [4, 7]: one study reported obtaining approximately 35 mg of native oANG from 1 L of plasma from a nephrectomized sheep [7]. As an alternative to native oANG, we prepared recombinant oANG using a Chinese hamster ovary (CHO) cell line that permanently expresses this protein [8], with a yield of approximately 8 mg purified oANG per liter of culture medium. Although sheep and a CHO cell line are effective sources for preparing milligram quantities of oANG, an animal and cell culture equipment, respectively, are required to produce oANG. Moreover, about one month of cell culture is required to obtain such amounts of recombinant oANG. Biotechnological advances that make it possible to prepare a large amount of oANG should facilitate clinical studies on renin.

To increase the availability of oANG, we aimed to produce recombinant oANG using an *Escherichia coli* expression system, which makes it possible to express the protein in a few days. Here, we report an *E. coli*-based system for the production of milligram levels of recombinant oANG and show that recombinant oANG expressed in *E. coli* functions as a human renin substrate.

## Results

### Expression screening of recombinant oANG in *E. coli*

**Fig. 1:**
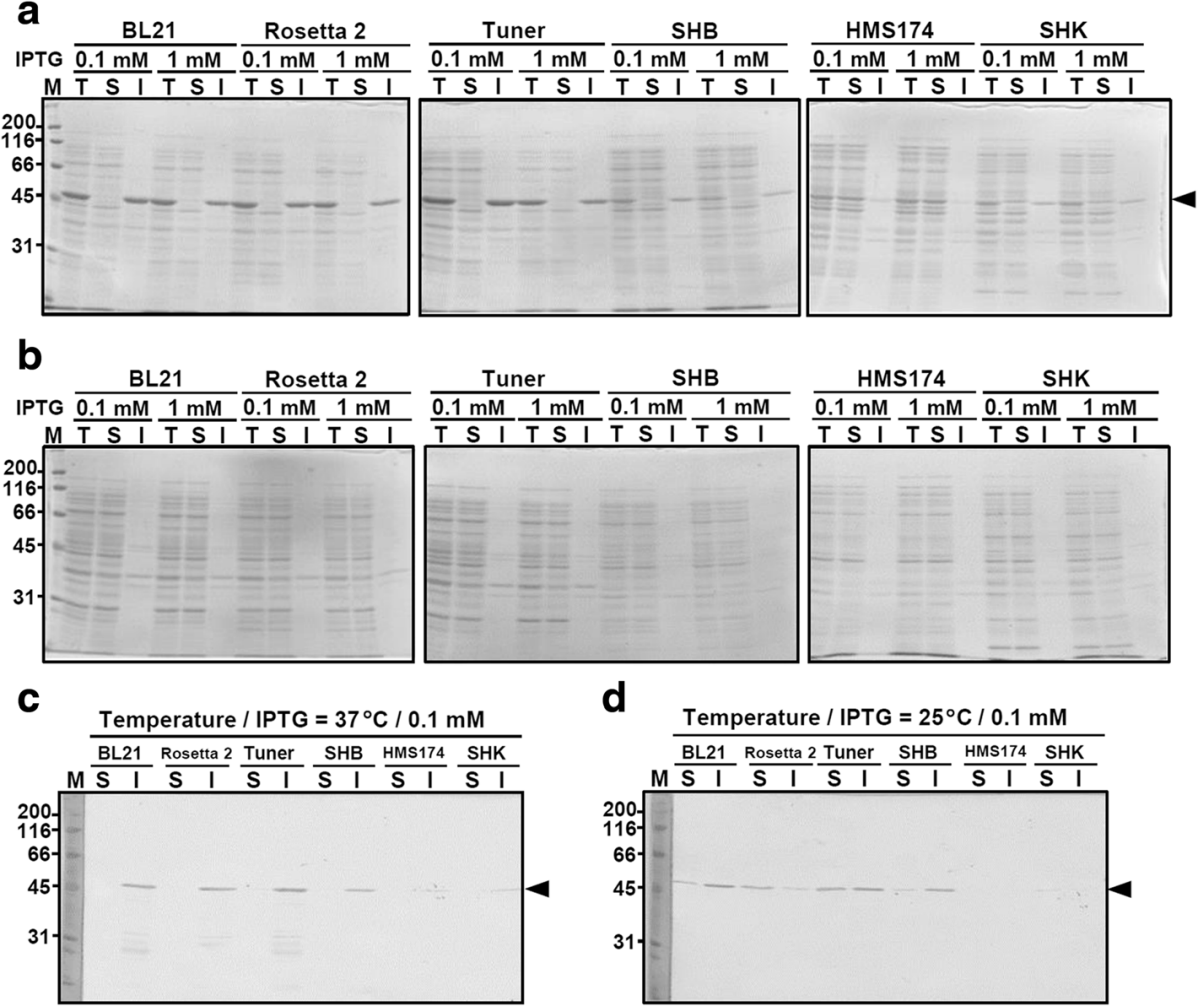
Expression screening of recombinant oANG in *E. coli.* Comparison of oANG expression and solubility using different *E. coli* (DE3) host strains and IPTG concentrations at 37 °C (**a**) and 25 °C (**b**). Recombinant oANG was expressed from pET-11a-oANG in *E. coli* (DE3) lysogens following induction with IPTG (0.1 and 1.0 mM), separated by SDS-polyacrylamide gel electrophoresis (SDS-PAGE), and then stained with Coomassie Brilliant Blue (CBB). Lane M, molecular marker; lane T, total cell lysate; lane S, soluble fraction; lane I, insoluble fraction. Western blot analysis of oANG expressed at 37 °C (**c**) and 25 °C (**d**) induced with IPTG (0.1 mM). Soluble (S) and insoluble (I) fractions of the cell lysates were analyzed by western blotting with an anti-oANG polyclonal antibody. Molecular markers (M) were visualized by CBB staining. The molecular weights of the marker proteins are shown on the left. The arrowhead on the right shows the size of oANG

As a first step towards expressing recombinant oANG in *E. coli*, we compared the effects of different host strains, induction levels, expression temperatures, and promoters on the solubility of oANG. Recombinant oANG was first expressed from an isopropyl-beta-D-thiogalactoside (IPTG)-inducible T7 promoter at 37 °C in DE3 lysogens of six tested *E. coli* strains. Under these conditions, oANG was found in the insoluble fraction of the cell lysate (Fig. 1a and c). We also found that *E. coli* B strains produced more oANG than *E. coli* K strains (Fig. 1a and c). When we reduced the expression temperature to 25 °C, no significant band corresponding to oANG was detected on a CBB stained SDS-polyacrylamide gel (Fig. 1b). Western blotting showed that when expressed at 25 °C, a small portion of the total oANG was located in the soluble fraction of the BL21(DE3), Rosetta 2(DE3), Tuner(DE3), and SHuffle T7 Express Competent (SHB) *E. coli* cell lysates (Fig. 1d). These results suggest that reducing the expression temperature facilitated proper folding, which yielded a higher amount of soluble oANG.

**Fig. 2:**
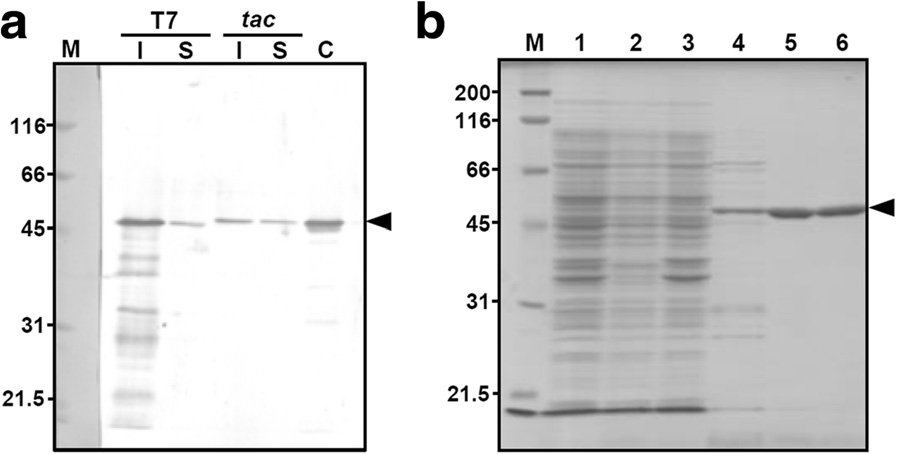
SDS-PAGE analysis of recombinant oANG expressed from a *tac* promoter. **a** Comparison of recombinant oANG expressed from either a T7 promoter or a *tac* promoter. Recombinant oANG expression was induced with IPTG (0.1 mM) at 37 °C. *E. coli* cells transformed with either pET-11a-oANG (T7 promoter) or pTAC-oANG-His (*tac* promoter) were diluted to a similar OD_600_ value and disrupted by sonication. Recombinant oANG in the soluble (S) and insoluble (I) fractions was visualized by western blotting. Lane C, control preparation of oANG produced by pTAC-oANG-His. **b** SDS-PAGE analysis of the purified recombinant oANG preparations. The pooled fractions after each purification step were separated by SDS-PAGE and stained with CBB. Lane M, molecular weight marker; lane 1, cell lysate; lane 2, supernatant of cell lysate; lane 3, precipitant of cell lysate; lane 4, pooled fractions after Ni-NTA Superflow Cartridge column chromatography; lane 5, after HiTrapQ HP column chromatography; lane 6, after HiLoad 16/60 Superdex 200 pg column chromatography. Molecular weights of the marker proteins are shown on the left. Arrowhead on the right shows the size of oANG

The *tac* promoter is a hybrid of the *lac* and *trp* promoters, and its transcriptional activity is weaker than that of the T7 promoter [9]. To produce soluble oANG at high yield, we expressed His-tagged oANG from an IPTG-inducible *tac* promoter at 37 °C in *E. coli* BL21 cells. To examine the amount of soluble oANG, we purified it from the supernatant of the cell lysate using Ni-affinity chromatography. The bound fraction contained a protein band with an estimated molecular weight that is similar to that of oANG (Additional file 1). This protein was identified as oANG by peptide mass fingerprinting using matrix-assisted laser desorption/ionization time-of-flight mass spectrometry. Next, we compared the effect of promoters on the solubility of recombinant oANG. When expressed from a T7 promoter, oANG accumulated in the insoluble fraction, and only a small proportion of oANG was located in the soluble fraction (Fig. 2a). In contrast, the soluble to insoluble ratio was approximately 1:1 when oANG was expressed from the *tac* promoter (Fig. 2a). These results indicate that expression from a weaker promoter (i.e., the *tac* promoter) increased the ratio of soluble to insoluble recombinant oANG.

### Purification of recombinant oANG expressed in *E. coli*

To prepare a larger amount of oANG, we harvested cell pellets (wet weight: 45 g) from a 15-L batch culture of *E. coli* BL21 cells harboring pTAC-oANG-His. After three successive chromatography steps, including a Ni-affinity column, an ion-exchange column, and a gel filtration column, approximately 0.27 mg of purified His-tagged oANG was obtained per liter of culture. When reacted with human renin, this preparation produced AI, indicating that the purified oANG is a functional renin substrate.

To increase the yield of soluble, active oANG, we utilized both auto-induction culture medium and a novel culture system to express His-tag-fused oANG from a *tac* promoter at 30 °C in *E. coli* BL21 cells. The absorbance of the culture medium at 600 nm (OD_600_) was approximately 15, indicating the high efficiency of this culture system. Using the three chromatographic steps described above, oANG was purified to homogeneity according to SDS-PAGE (Fig. 2b). Its molecular weight was estimated to be 50 k, which is similar to its calculated molecular mass (50.3 kDa).

The specific amount in the final preparation was 23.5 μg of AI/mg of total protein. Assuming a molecular mass of 50.3 kDa for oANG and that one mole of oANG yields one mole of AI with a molecular mass of 1.296 kDa, the theoretical specific amount of ANG is 25.8 μg of AI/mg. Based on this value, the purity of the final preparation was 91 %. The production yield was approximately 4.0 mg of purified oANG per liter of culture, although 0.5 L of culture (cell pellet wet weight: 8.7 g) was routinely used for oANG production. On the other hand, the production yield using CHO cells was approximately 2.3 mg of purified oANG per liter of culture. In the following analyses, recombinant oANG expressed in *E. coli* (oANG_Ecoli_) was compared with that expressed in CHO cells (oANG_CHO_).

### Protein size analysis of recombinant oANG

**Fig. 3:**
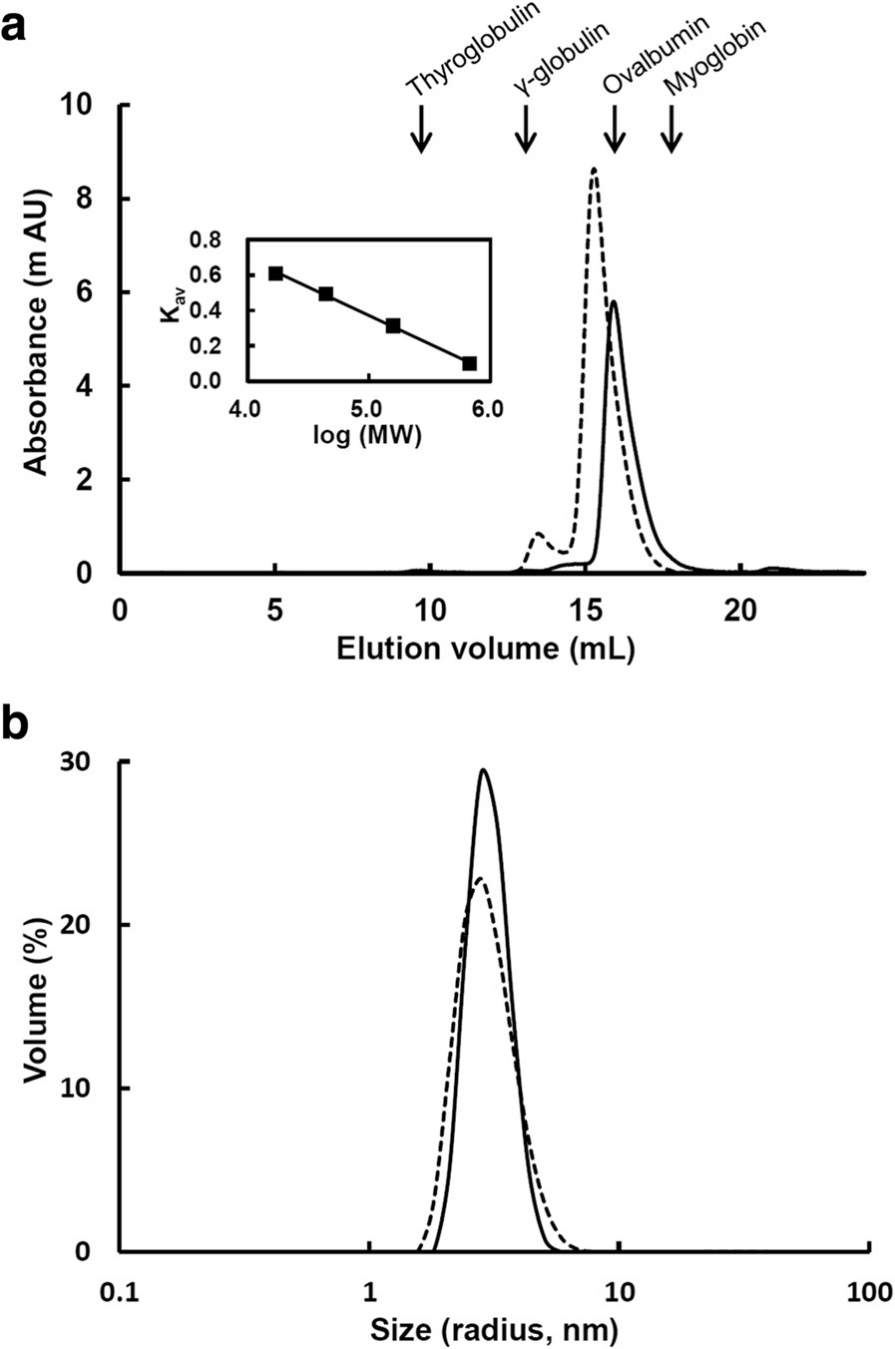
Protein size analysis of recombinant oANG_Ecoli_ and oANG_CHO_. The sizes of oANG_Ecoli_ (solid line) and oANG_CHO_ (dotted line) were determined by analytical size-exclusion gel filtration (**a**) and DLS (**b**). Purified oANG was loaded onto a Superdex 200 10/300 GL column equilibrated with 2 mM HEPES and 0.20 M KCl (pH 8.0). The elution volumes of the marker proteins are shown. The calibration curve is shown in the inset. DLS data were measured at 25 °C in a pH 7.4 buffer solution containing 0.5 mg/mL oANG, 20 mM sodium phosphate, and 20 mM (oANG_Ecoli_) or 33 mM (oANG_CHO_) KCl. The size distribution by percent volume is presented

To examine the molecular size of recombinant oANG, we used analytical size-exclusion gel filtration and dynamic light scattering (DLS). The results of size-exclusion gel filtration showed that both recombinant oANG_Ecoli_ and oANG_CHO_ exhibited one major peak (Fig. 3a). The apparent molecular weight of recombinant oANG_Ecoli_ was 42 k. In contrast, recombinant oANG_CHO_ eluted more quickly (Fig. 3a), and its apparent molecular weight was 56 k. The DLS results (Fig. 3b) showed that recombinant oANG_Ecoli_ and oANG_CHO_ displayed one size distribution, with 19.1 and 26.5 % polydispersity, respectively. The hydrodynamic radii of recombinant oANG_Ecoli_ and oANG_CHO_ were estimated to be 3.05 ± 0.58 nm and 3.02 ± 0.80 nm, respectively, corresponding to globular proteins with molecular masses of 45.7 kDa and 44.8 kDa, respectively. As the calculated molecular mass is 50.3 kDa (see *Methods*), these results show that recombinant oANG is a monomer in solution. This finding is consistent with our previous report on oANG_CHO_ [10].

### Protein structure and stability analysis of recombinant oANG

**Fig. 4:**
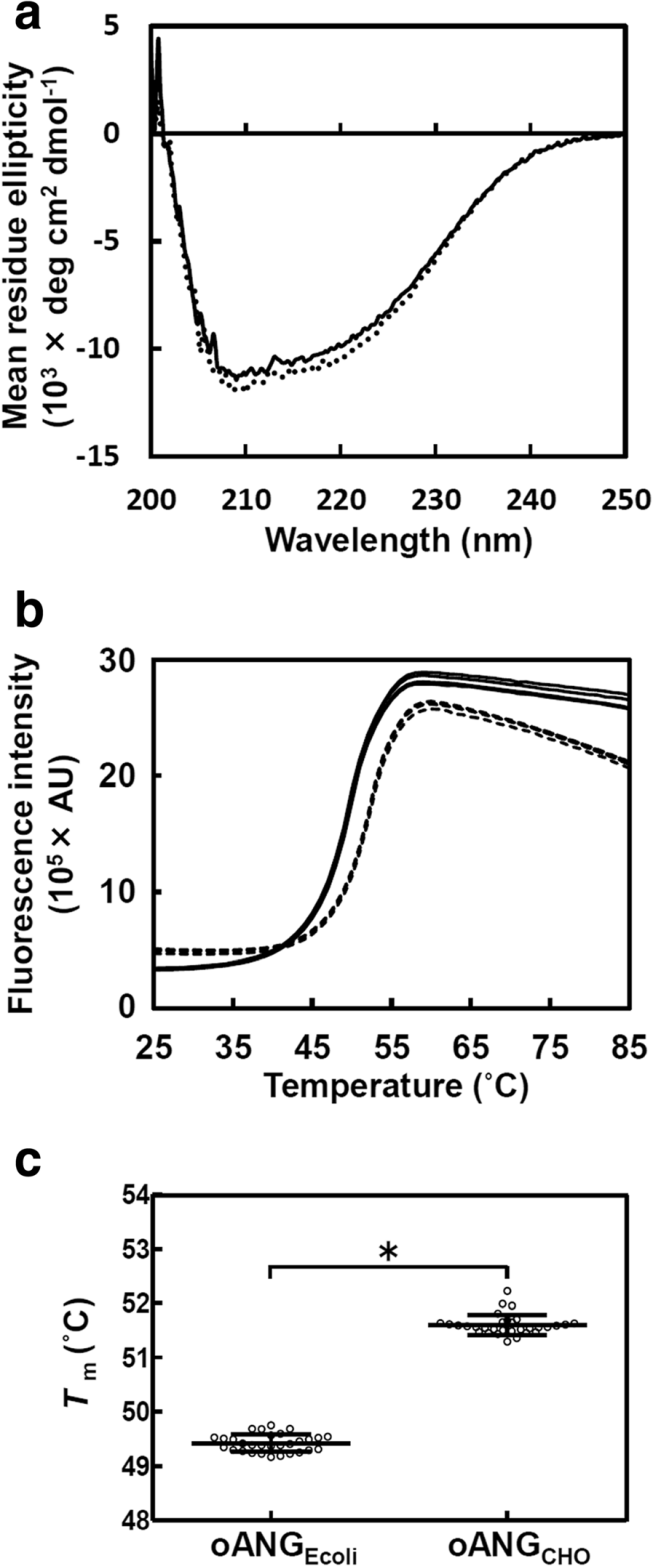
Protein structure and stability analysis of recombinant oANG_Ecoli_ and oANG_CHO._ Purified oANG_Ecoli_ (*solid line*) and oANG_CHO_ (*dotted line*) were analyzed by CD (**a**) and DSF (**b**). CD data were measured at 25 °C in a pH 7.4 buffer solution containing 0.5 mg/mL oANG, 20 mM sodium phosphate, and 20 mM (oANG_Ecoli_) or 33 mM (oANG_CHO_) KCl. DSF data were measured with 10 μM oANG in 5× SYPRO Orange, 200 mM HEPES, and 0.15 M KCl (pH 7.5). Five typical plots for each preparation are shown. **c** Comparison of the *T*_m_ of oANG_Ecoli_ and oANG_CHO_. *T*_m_ values are plotted as open circles. The horizontal line and error bars are the mean value and standard deviation of the *T*_m_ values, respectively (oANG_Ecoli_, *N* = 30; oANG_CHO_, *N* = 31). *P < 0.0001 by Student’s *t*-test

Circular dichroism (CD) analysis was carried out to obtain secondary structural information for recombinant oANG. The CD spectrum of recombinant oANG_Ecoli_ was similar to that of oANG_CHO_ (Fig. 4a). Recombinant oANG_Ecoli_ was estimated to be composed of 24 % alpha helix and 22 % beta strand. These values are similar to those calculated from the crystal structure of human angiotensinogen [PDB ID: 2WXW], which is composed of 28 % alpha helix and 23 % beta strand.

To examine the stability of recombinant oANG, we used differential scanning fluorimetry (DSF). This method monitors the thermal unfolding of proteins in the presence of a hydrophobic fluorescent dye, and then the fluorescence intensity is plotted as a function of temperature [11]. The resulting intensity plot is used to estimate the melting temperature (*T*_m_). The DSF results (Fig. 4b) showed that both recombinant oANG products (CHO and *E. coli*) undergo a two-state transition during the course of thermal denaturation. The *T*_m_ of recombinant oANG_Ecoli_ and oANG_CHO_ was 49.4 ± 0.16 °C and 51.6 ± 0.19 °C, respectively (Fig. 4c).

### Human renin reactivity

**Fig. 5:**
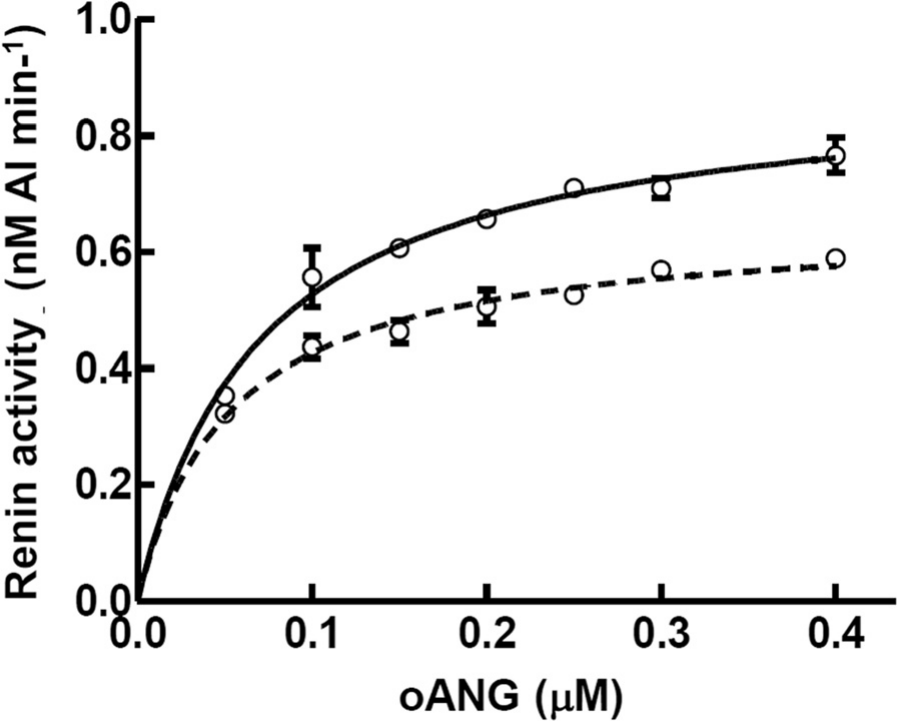
Kinetic analysis of human renin with recombinant oANG_Ecoli_ and oANG_CHO_. Renin activity in the presence of oANG_Ecoli_ (*solid line*) and oANG_CHO_ (*dotted line*) was analyzed by steady-state enzyme kinetics. Various concentrations of oANG were incubated with human renin (25 pM) at 37 °C in sodium phosphate buffer (pH 7.2) containing DFP and EDTA. The amount of AI produced was measured by enzyme-linked immunosorbent assay (ELISA). The error bars represent the standard deviation of the velocities (*N* = 3)

To determine whether the catalytic efficiency of recombinant oANG_Ecoli_ is comparable to oANG_CHO_, we carried out a steady-state kinetic analysis using both oANG preparations. Both exhibited a typical substrate saturation curve (Fig. 5). The Michaelis constant (*K*_m_) values of the two preparations were similar, whereas the catalytic turnover (*k*_cat_) of oANG_Ecoli_ was slightly higher than that of oANG_CHO_ (Table 1). The order of magnitude of the catalytic efficiency (*k*_cat_/*K*_m_) for the two preparations is in good agreement with that of a previous study by Nakagawa et al. [12].

**Table 1:** Kinetic parameters of recombinant oANG_Ecoli_ and oANG_CHO_

oANG	*K*_m_ (μM)	*k*_cat_ (s^−1^)	*k*_*cat*_ */K*_*m*_ (M^−1^ s^−1^)
oANG_Ecoli_^a^	0.071	0.59	8.3 × 10^6^
oANG_CHO_^a^	0.072	0.46	6.4 × 10^6^
oANG_CHO_^b^	0.090	0.31	3.4 × 10^6^

^a^This work

^b^Nakagawa et al. [12]

## Discussion

ANG has significant sequence homology to representative members of the serine protease inhibitor (serpin) superfamily [13]. Various recombinant serpins have been produced in *E. coli* [14]. When expressed in *E. coli*, some serpins are soluble, while others become insoluble and require refolding [14]. Kunapuli et al. [15] purified human ANG expressed from an IPTG-inducible *lac* promoter in *E. coli*. This preparation was cleaved by human kidney renin, as assessed by SDS-PAGE and western blotting; however, its enzymatic characterization was not reported [15]. Zou et al. [16] expressed recombinant human, mouse, and rat ANGs in *E. coli* using a SUMO-fusion system. According to the data deposited in the Protein Data Bank [PDB ID: 2WXW], a pSUMO3 plasmid with a T7 promoter was used to express human ANG. The authors characterized the enzymatic properties of human ANG and successfully determined the crystal structures of human, mouse, and rat ANG as well as a renin-ANG complex [16]. This report shows that the combination of *E. coli* and the SUMO-fusion system is a good choice for expressing recombinant ANG; however, the details of the expression and purification conditions are not available [16]. In the present study, a procedure was developed to prepare milligram quantities of recombinant oANG expressed in *E. coli*.

When recombinant oANG was expressed from a T7 promoter at 37 °C in various *E. coli* strains, it accumulated in the insoluble fraction (Fig. 1a and c). The breakthroughs in this study were (*a*) the use of a *tac* promoter and (*b*) the combination of a novel culture system and auto-induction culture medium. The former and the latter were shown to be useful in previous reports by Ikeda-Boku et al. [17] and Li et al. [18], respectively. When the protein was expressed from a *tac* promoter at 37 °C, the ratio of soluble to insoluble recombinant oANG increased significantly (Fig. 2a), and 4.0 mg of recombinant oANG was prepared from a 15-L culture of *E. coli*. Although a milligram amount of oANG was obtained using this procedure, the production yield was relatively low (0.27 mg of oANG per liter of culture). With an aim to develop a higher-yield method, we devised a novel culture system in which *E. coli* cells were grown in culture medium that was aerated and agitated in a glass bottle. In addition, we used auto-induction medium [19] in which *E. coli* cells initiate recombinant protein expression from a *lac*-based promoter when they switch carbon sources from glucose to lactose. As a result, the production yield increased by a factor of 15, from 0.27 mg to 4.0 mg of oANG per liter of culture. These breakthroughs enabled us to prepare a milligram quantity of oANG on a small culture scale.

We compared the biophysical and enzymatic properties of recombinant oANG_Ecoli_ and oANG_CHO_. The proteins in both oANG preparations are a similar size (as estimated by DLS; Fig. 3b) and have similar secondary structural elements (Fig. 4a), a two-state transition during thermal denaturation (Fig. 4b), a similar affinity for human renin (*K*_m_ value, Table 1), and a similar catalytic efficiency (*k*_cat_/*K*_m_, Table 1). These results strongly suggest that the three-dimensional structure of recombinant oANG_Ecoli_ is very similar to that of oANG_CHO_. Although the transcription, translation, and post-translational modifications of proteins differ between *E. coli* and CHO cells, recombinant oANG_Ecoli_ must be properly folded since, according to Anfinsen’s dogma [20], the amino acid sequence of a protein is sufficient to determine its three-dimensional fold. In summary, these results show that recombinant oANG expressed in *E. coli* is functionally comparable to oANG expressed in CHO cells.

Recombinant oANG_CHO_ possesses a single *N*-linked oligosaccharide chain [10]. On the other hand, recombinant oANG_Ecoli_ does not have the oligosaccharide chain because standard *E. coli* cells do not attach oligosaccharide chains to *N*-linked glycosylation sites [21]. The absence of the *N*-linked oligosaccharide on oANG_Ecoli_ may be the cause of its lower *T*_m_ value (Fig. 4c).

Compared in terms of “production yield divided by expression days,” the *E. coli*-based and CHO-based production systems produced 2.0 and 0.077 mg oANG/liter of culture/day of culture, respectively. Therefore, the *E. coli*-based production system developed in this study is 26 times more time efficient than the CHO-based system.

## Conclusions

We developed an *E. coli* expression system that enables the rapid and easy production of recombinant oANG protein and successfully prepared 4.0 mg of purified oANG per liter of culture medium. Recombinant oANG expressed in *E. coli* functions as a human renin substrate. Plasma renin concentration (PRC) is used as a clinical parameter related to hypertension [6]. This newly developed *E. coli* expression system makes human renin substrate more available, which will be useful for clinical studies on renin.

## Methods

### Construction of expression plasmid

The *oANG* gene was amplified by polymerase chain reaction (PCR) from a pcDNA3 mammalian expression vector harboring oANG cDNA [8]. The sequences of the forward and reverse primers were 5′-ATATATATcatatgGACCGCGTATACATCCACCCCTTCCACCTC-3′ (NdeI site in lower case) and 5′-ATTTggatccTTATTACTCAGCGCTCAGCGGGCG-3′ (BamHI site in lower case), respectively. The pET-11a vector (Novagen, Madison, WI) and the amplified DNA fragment containing *oANG* were digested with NdeI and BamHI and ligated to generate pET-11a-oANG. The encoded protein comprises 453 amino acids residues (49.2 kDa), including the mature angiotensinogen (D^25^-E^476^) of oANG [GenBank: BAA04470] [22] and an N-terminal M extension (derived from the initiation codon).

To produce an oANG expression plasmid with the protein under the control of the *tac* promoter, a pTAC-MAT-Tag vector (Sigma-Aldrich, St. Louis, MO) was modified by adding a new restriction enzyme site to the multiple cloning site. The *oANG* gene was amplified by PCR from pET-11a-oANG. The sequences of the forward and reverse primers were 5′-GGGGTCTAGAcatatgGACCGCGTATACATCCACCCCTTC-3′ (NdeI site in lower case) and 5′-GGGGAAGCTTctcgagCTCAGCGCTCAGCGGGC-3′ (XhoI site in lower case), respectively. The amplified fragment and the pTAC vector were digested with NdeI and XhoI. The DNA fragment containing *oANG* was sub-cloned into the NdeI and XhoI sites of the pTAC vector to produce pTAC-oANG-His. The encoded protein comprises 461 amino acids residues (50.3 kDa), including the mature angiotensinogen (D^25^-E^476^) [22] as well as an N-terminal M extension (derived from the initiation codon) and a C-terminal extension (LEHHHHHH).

### Expression of recombinant oANG in *E. coli*

The pET-11a-oANG plasmid was used to transform six different *E. coli* host strains: BL21(DE3), Rosetta 2(DE3), Tuner(DE3), HMS174(DE3) (Novagen), SHuffle T7 Express Competent *E. coli* (SHB), and SHuffle T7 Competent *E. coli* (SHK) (New England Biolabs, Herts, UK). Ten randomly selected colonies were suspended in 0.05 mL of LB medium (Nacalai, Kyoto, Japan), and 0.02 mL of the suspensions were added to 2 mL of LB medium supplemented with either 50 μg/mL ampicillin [all host strains except for Rosetta 2(DE3)] or 50 μg/mL ampicillin and 25 μg/mL chloramphenicol [Rosetta 2(DE3)]. After the transformed cells were grown at 37 °C to mid-log phase (OD_600_ approximately 0.6), IPTG was added to the culture medium and protein expression was induced for 3 h. IPTG concentrations and expression temperatures were varied in an attempt to improve the solubility of oANG. Cells were collected by centrifugation (2,200 × *g*, 10 min, 4 °C), resuspended in phosphate-buffered saline (10 mM Na_2_HPO_4_, 1.8 mM KH_2_PO_4_, 140 mM NaCl, and 2.7 mM KCl, pH 7.3), and disrupted by sonication. The soluble and insoluble fractions were separated by centrifugation (13,200 × *g*, 15 min, 4 °C). All samples were analyzed by SDS-PAGE in 12 % gels using either CBB staining or western blotting with rabbit anti-oANG polyclonal anti-serum (1:10,000 dilution) as a primary antibody [8]. In the western blot, the antigen-antibody complex was tagged using an anti-rabbit secondary antibody conjugated with a horseradish peroxidase and was detected by a chromogenic reaction using hydrogen peroxide and 3,3′-diaminobenzidine.

To examine the effect of the promoter, BL21(DE3) cells and BL21 cells were transformed with pET-11a-oANG and pTAC-oANG-His, respectively. Ten randomly selected colonies of each transformant were cultured in 2 mL of LB medium at 37 °C, and recombinant oANG expression was induced at 37 °C by the addition of 0.1 mM IPTG for 3 h. The harvested cells were suspended in 1 mL of Buffer A (20 mM Tris-HCl, 0.3 M KCl, and 1 mM MgCl_2_, pH 7.6). The OD_600_ value of *E. coli* cells transformed with pET-11a-oANG (containing a T7 promoter) was 0.81, whereas that of cells transformed with pTAC-oANG-His (containing a *tac* promoter) was 0.79. After disrupting the *E. coli* cells by sonication, the soluble and insoluble fractions were prepared for western blotting.

### Expression of recombinant oANG in *E. coli*

*E. coli* BL21 cells were transformed with the pTAC-oANG-His plasmid. Ten randomly selected colonies were suspended and cultured in 2 mL of LB medium supplemented with 50 μg/mL ampicillin. After the transformed cells were grown at 37 °C to mid-log phase (OD_600_ approximately 0.6), a culture aliquot was inoculated into 0.5 L of Overnight Express Instant TB medium (Novagen) [19] supplemented with 50 μg/mL ampicillin, 1 % glycerol, and 0.01 % Antifoam SI (Wako, Osaka, Japan). The cells were grown for 16 h at 30 °C using a novel culture system, aerated at 1 volume of air per unit volume of medium per min, and agitated at 600 rpm with a magnetic stirrer bar (ϕ 8 mm, length 60 mm) in a 2-L glass bottle. Cells were collected by centrifugation (10,000 × *g*, 10 min, 4 °C) using a high-speed refrigerated centrifuge (Model 6000; Kubota, Japan). The cell pellets were stored at -80 °C until use. About 2 days were required for this *E. coli*-based expression of oANG.

### Purification of recombinant oANG expressed in *E. coli* (oANG_Ecoli_)

Approximately 8 g of *E. coli* cells were suspended in 100 mL of Buffer A. After the addition of 3 μL of 30 KU/μL rLysozyme (Novagen) and 3 μL of 25 U/μL Benzonase (Novagen), the cell suspension was incubated on ice for 30 min with gentle stirring. The cells were disrupted by sonication using an ultrasonic homogenizer (Microson XL2000; MISONIX). The cell lysate was centrifuged at 14,000 × *g* for 30 min at 4 °C, and the resulting supernatant was centrifuged again at 20,000 × *g* for 15 min at 4 °C. The clear supernatant was loaded onto a 1-mL Ni-NTA Superflow Cartridge column (Qiagen, Venlo, The Netherlands) equilibrated with Buffer A on an ÄKTAprime plus (GE Healthcare, Buckinghamshire, England). After washing with 0.01 M imidazole in Buffer A, proteins were eluted with a step-wise gradient (0.01 M–0.2 M) of imidazole in Buffer A. The Ni-NTA bound fractions were pooled, diluted 1:10 with 20 mM Tris-HCl (pH 8.0), and loaded onto a HiTrapQ HP column (GE Healthcare) equilibrated with the same buffer. Proteins were eluted with a linear gradient (0.02 M–0.4 M) of KCl in the same buffer. Bound fractions were pooled, concentrated using a Vivaspin Turbo 15 with a molecular cut-off of 10,000 (Sartorius, Goettingen, Germany), and subjected to gel filtration on a HiLoad 16/60 Superdex 200 pg column (GE Healthcare) equilibrated with 2 mM 2-[4-(2-hydroxyethyl)-1-piperazinyl]ethanesulfonic acid (HEPES) and 0.2 M KCl (pH 8.0). The oANG-containing fractions were pooled and concentrated to 5.0 mg/mL using a Vivaspin Turbo 15. The molar concentration of oANG was determined using the molecular extinction coefficient at 280 nm (39,165 M^−1^ cm^−1^), which was calculated according to the formula provided by Kuramitsu et al. [23].

### Preparation of recombinant oANG expressed in CHO cells (oANG_CHO_)

Recombinant oANG was expressed in CHO cells and purified as described previously [8, 24]. About 30 days were required for the CHO cell-based expression of oANG. Approximately 0.8 L of serum-free culture medium was collected. Ammonium sulfate was added to the cleared culture medium to 70 % saturation. After removing most of the supernatant, the precipitate was collected by centrifugation (10,000 × *g*, 10 min, 4 °C). The precipitate was dissolved in 20 mM sodium acetate (pH 5.0) and dialyzed against the same buffer. The dialysate was centrifuged at 10,000 × *g* for 10 min at 4 °C to remove the insoluble material. The clear supernatant was loaded onto a HiTrap SP FF column (GE Healthcare) equilibrated with 20 mM sodium acetate (pH 5.0). oANG was eluted with a linear gradient of NaCl (0–1.0 M) in the same buffer. The bound fractions were pooled, concentrated using a Vivaspin Turbo 15, and subjected to gel filtration on a HiLoad 16/60 Superdex 200 pg column (GE Healthcare) equilibrated with 2 mM HEPES and 0.2 M KCl (pH 8.0). The oANG-containing fractions were pooled and concentrated to 3.0 mg/mL using a Vivaspin Turbo 15. The above molecular extinction coefficient at 280 nm was used to estimate the molar concentration of oANG.

### Preparation of recombinant human renin

Recombinant human prorenin was expressed in CHO cells and purified as described previously [5]. Approximately 1 L of serum-free culture medium was collected. Proteins in the cleared medium were precipitated with ammonium sulfate at 75 % saturation. The precipitate was dissolved in 20 mM acetate (pH 5.0) and dialyzed against the same buffer. Human prorenin was purified on a Resource S column (GE Healthcare) with a linear gradient of NaCl (0–1.0 M) in 20 mM acetate (pH 5.0). Immediately after purification, fractions containing human prorenin were mixed with one-tenth volume of 0.1 M Tris (pH 8) to neutralize the pH. The protein concentration of human prorenin was determined using a molecular extinction coefficient at 280 nm of 47,705 M^−1^cm^−1^, which was calculated with the ProtParam tool [25], and the final concentration of human prorenin was 2.4 μM. Human prorenin was treated with trypsin to prepare human renin, as described previously [26].

### Protein assay

Total protein concentration was measured as described by Bradford [27] using bovine serum albumin as a standard.

### Angiotensinogen assay

The concentration of recombinant oANG was determined as described previously [8]. Recombinant oANG was incubated with excess human renin at 37 °C for 30 and 45 min in 5 mM sodium phosphate buffer (pH 7.2) containing 5 mM diisopropyl fluorophosphates (DFP), 5 mM EDTA, 100 mM NaCl, and 0.1 % (*w/v*) heat-denatured bovine serum albumin (fraction V). The amount of AI generated was measured in triplicate by AI-ELISA [28]. The plateau amount of AI generated was represented as the concentration of recombinant oANG.

### Size-exclusion gel filtration analysis

Purified oANG was subjected to gel filtration on a Superdex 200 10/300 GL column (GE Healthcare) equilibrated with 2 mM HEPES and 0.20 M KCl (pH 8.0) at a flow rate of 0.3 mL/min. The column was calibrated using the Gel Filtration Standard (Bio-Rad Laboratories, Hercules, CA). The calibration curve is represented by the equation K_av_ = 1.9754 - 0.3211(log_10_Mw), where K_av_ is the gel-phase distribution coefficient and Mw is the molecular weight of the protein.

### DLS analysis

The DLS experiments were performed at 25 °C with a Zetasizer Nano-ZS (Malvern, Worcestershire, UK). Recombinant oANG preparations were diluted to a final concentration of 0.5 mg/mL with 20 mM sodium phosphate (pH 7.4), and the oANG_Ecoli_ and oANG_CHO_ preparations contained 20 and 33 mM KCl, respectively. Prior to the measurements, the samples were filtered (Ultrafree-MC, 0.22 μm, Millipore). The polydispersity, hydrodynamic radius, and empirically estimated molecular mass were calculated using the Zetasizer software provided by the manufacturer. The hydrodynamic radius was reported as the mean ± standard deviation. The viscosity and refractive index values of the solvent used were 0.9056 cP and 1.331, respectively, as calculated by the software.

### CD spectroscopic analysis

CD spectra were recorded at 25 °C on a J-820 spectropolarimeter (Jasco International, Tokyo, Japan) equipped with a Peltier thermoregulation system using 1-mm thick quartz cells (S11-UV-10; GL Sciences). Recombinant oANG preparations were diluted to a final concentration of 0.5 mg/mL with 20 mM sodium phosphate (pH 7.4), and the oANG_Ecoli_ and oANG_CHO_ preparations contained 20 and 33 mM KCl, respectively. Prior to the measurements, the samples were filtered (Ultrafree-MC, 0.22 μm, Millipore). CD spectra between 190 and 260 nm were measured with a scanning speed of 50 nm/min and a data pitch of 0.2 nm. Spectra from eight scans were averaged. The contribution of buffer was subtracted from each spectrum. Mean residue ellipticity values were calculated assuming that the number of residues in oANG_Ecoli_ and oANG_CHO_ was 461 and 452, respectively. The experimental data in the 200 nm–240 nm range were analyzed by the K2D3 web server [29] to estimate the secondary structure content.

### DSF analysis

DSF experiments were performed as described previously [11]. Recombinant oANG preparations were diluted in 200 mM HEPES and 0.15 M KCl (pH 7.5) to a final concentration of 20 μM. The fluorescent dye SYPRO Orange (Life Technologies, Carlsbad, CA) was diluted to 10× (from a 5000× stock). Recombinant oANG (20 μM) and SYPRO Orange (10×) were mixed in a 1:1 ratio. The mixture was aliquoted into a 96-well PCR plate and sealed with optical quality sealing film (Applied Biosystems, Foster City, CA). Thermal unfolding was carried out using a StepOnePlus real-time PCR instrument (Applied Biosystems) by heating from 25 to 95 °C at a rate of 1 °C per min. Fluorescence intensity was measured every 1 °C using a ROX filter at excitation and emission wavelengths of 490 and 600 nm–630 nm, respectively. Fluorescence intensities in relative fluorescence units were plotted as a function of temperature. To estimate the *T*_m_, the Boltzmann equation was used to fit the fluorescence data using GraphPad Prism 5.0 software (GraphPad Software, La Jolla, CA) and the calculation templates downloaded from ftp://ftp.sgc.ox.ac.uk/pub/biophysics/ [11]. The *T*_m_ values were reported as the mean ± standard deviation. Student’s *t*-test was used to statistically analyze the results.

### Kinetic analysis

Various concentrations (0–0.4 μM) of recombinant oANG were incubated with human renin (25 pM) at 37 °C for 30 min in 5 mM sodium phosphate buffer (pH 7.2) containing 8 mM DFP, 8 mM EDTA, 100 mM NaCl, and 0.1 % (w/v) heat-denatured bovine serum albumin (fraction V). The rate of AI generation was determined by AI-ELISA [28]. The *K*_m_ and maximum velocity (*V*_max_) were estimated by Hanes-Woolf plot using GraphPad Prism 5.0 software.

### Availability of data and material

The datasets supporting the conclusions of this article are included within the article and its additional file.

## Additional file

Additional file 1: Purification of recombinant oANG using Ni-affinity column chromatography. (PDF 64 kb)

## Abbreviations

AI: angiotensin I
ANG: angiotensinogen
CBB: Coomassie Brilliant Blue
CD: circular dichroism
CHO: Chinese hamster ovary
DFP: diisopropyl fluorophosphates
DLS: dynamic light scattering
DSF: differential scanning fluorimetry
ELISA: enzyme-linked immunosorbent assay
HEPES: 2-[4-(2-hydroxyethyl)-1-piperazinyl]ethanesulfonic acid
IPTG: isopropyl-beta-D-thiogalactoside
*k*_cat_: catalytic turnover
*k*_cat_/*K*_m_: catalytic efficiency
*K*_m_: Michaelis constant
oANG: ovine ANG
oANG_CHO_: ovine angiotensinogen expressed in CHO cells
oANG_Ecoli_: ovine angiotensinogen expressed in *E. coli*
PRC: plasma renin concentration
SDS-PAGE: SDS-polyacrylamide gel electrophoresis
serpin: serine protease inhibitor
SHB: SHuffle T7 Express Competent *E. coli*
SHK: SHuffle T7 Competent *E. coli*
*T*_m_: melting temperature
