# Supplementary material for: Escherichia coli-based production of recombinant ovine angiotensinogen and its characterization as a renin substrate
Source: BMC Biotechnol. 2016 Apr 7;16:33. doi: 10.1186/s12896-016-0265-x (PMC4823841; doi:10.1186/s12896-016-0265-x)
Supplement: Additional file 1: — Purification of recombinant oANG using Ni-affinity column chromatography. (PDF 64 kb) [file 12896_2016_265_MOESM1_ESM.pdf]

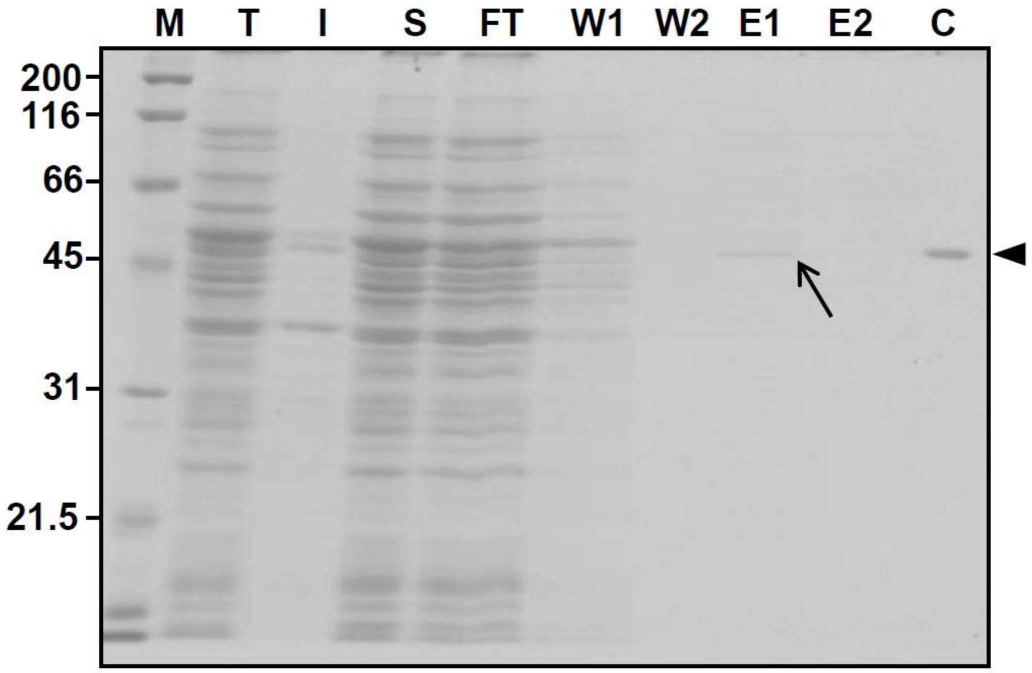

Additional file 1. Purification of recombinant oANG using Ni-affinity column chromatography.

After recombinant oANG was expressed from a *tac* promoter in *E. coli* cells, the cell pellet was disrupted by sonication in Tris buffer (pH 7.6). The resulting total cell lysate (T) was centrifuged to produce the insoluble (I) and soluble (S) fractions. The soluble fraction was subjected to metal affinity column chromatography using Ni-NTA agarose. FT, flowthrough; W1 and W2, washes; E1 and E2, elution fractions (with an imidazole concentration of 0.5 M); and C, control preparation of oANG. All preparations were analyzed by SDS-PAGE (12% gel, CBB staining). Molecular weights of the marker proteins are shown on the left. Arrowhead on the right shows the size of oANG. The protein band in lane E1, with a molecular weight similar to that of oANG, is shown by an arrow. This result showed that the bound fraction contained a protein band with an estimated molecular weight that is similar to that of oANG.
